# Estimated costs for the delivery of safer conception strategies for HIV-discordant couples in Zimbabwe: a cost analysis

**DOI:** 10.1186/s12913-020-05784-4

**Published:** 2020-10-12

**Authors:** Carolyn Smith Hughes, Joelle Brown, Caroline Murombedzi, Thandiwe Chirenda, Gift Chareka, Felix Mhlanga, Bismark Mateveke, Serah Gitome, Tinei Makurumure, Allen Matubu, Nyaradzo Mgodi, Zvavahera Chirenje, James G. Kahn

**Affiliations:** 1 Institute for Global Health Sciences, University of California, 550 16th Street, 3rd Floor, San Francisco, 94158 USA; 2 Department of Obstetrics, Gynecology and Reproductive Sciences, University of California, San Francisco, USA; 3 Department of Epidemiology and Biostatistics, University of California, San Francisco, USA; 4 College of Health Sciences Clinical Trials Research Centre, University of Zimbabwe, Harare, Zimbabwe; 5 Harare Central Hospital, Harare, Zimbabwe; 6 Centre for Microbiology Research, Kenya Medical Research Institute, Nairobi, Kenya; 7 Mercy-Care Fertility Centre, Harare, Zimbabwe; 8 Philip R. Lee Institute for Health Policy Studies, University of California, San Francisco, San Francisco, USA

**Keywords:** HIV, Conception, Cost, ART, PrEP, Semen-washing, Discordant

## Abstract

**Background:**

In recent years, safer conception strategies have been developed to help HIV-serodiscordant couples conceive a child without transmitting HIV to the seronegative partner. The SAFER clinical trial assessed implementation of these strategies in Zimbabwe.

**Methods:**

As a part of the SAFER study, we estimated the costs (in 2017 $US) associated with individual and combination strategies, in the trial setting and real-world practice, from a healthcare system perspective. Safer conception strategies included: 1) ART with frequent viral load testing until achieving undetectable viral load (ART-VL); 2) daily oral pre-exposure prophylaxis (PrEP); 3) semen-washing with intrauterine insemination; and 4) manual self-insemination at home. For costs in the trial, we used a micro-costing approach, including a time and motion study to quantify personnel effort, and estimated the cost per couple for individual and combination strategies for a mean of 6 months of safer services. For real-world practice, we modeled costs for three implementation scenarios, representing differences from the trial in input prices (paid by the Ministry of Health and Child Care [MOHCC]), intervention intensity, and increments to current HIV prevention and treatment practices and guidelines. We used one-way sensitivity analyses to assess the impact of uncertainty in input variables.

**Results:**

Individual strategy costs were $769–$1615 per couple in the trial; $185–$563 if using MOHCC prices. Under the target intervention intensity and using MOHCC prices, individual strategy costs were $73–$360 per couple over and above the cost of current HIV clinical practices. The cost of delivering the most commonly selected combination, ART-VL plus PrEP, ranged from $166–$517 per couple under the three real-world scenarios. Highest costs were for personnel, lab tests, and strategy-specific consumables, in variable proportions by clinical strategy and analysis scenario. Total costs were most affected by uncertainty in the price of PrEP, number of semen-washing attempts, and scale-up of semen-washing capacity.

**Conclusions:**

Safer conception methods have costs that may be affordable in many low-resource settings. These cost data will help implementers and policymakers add safer conception services. Cost-effectiveness analysis is needed to assess value for money for safer conception services overall and for safer strategy combinations.

**Trial registration:**

Registry Name: Clinicaltrials.gov. Trial registration number: NCT03049176. Registration date: February 9, 2017.

## Background

With an estimated 14% prevalence of HIV among adults of age 15–49, Zimbabwe has been hit particularly hard by the HIV pandemic [1]. With advances in the development, evidence generation, and scale-up of antiretroviral therapy (ART) for HIV treatment, HIV-positive individuals are able to lead more “normal” lives, which includes having sexual relationships and conceiving children—activities that were previously discouraged and stigmatized in HIV-positive individuals [2]. Studies have shown a strong interest and intention for having children among HIV-positive adults in sub-Saharan African countries [3–5]. Safer conception strategies have been developed to help HIV-discordant couples (one partner has HIV while the other does not) conceive a child without transmitting HIV to the seronegative partner (and infant) [6, 7].

Though scale-up of ART for HIV-infected persons has been extensively studied in Zimbabwe, and the roll-out of PrEP for HIV prevention in high-risk populations is now underway [1–4], data on the estimated resources and costs associated with the delivery of safer conception strategies are lacking. Costs associated with the use of HIV prevention strategies in HIV-discordant couples who wish to conceive a child can help inform future plans to provide safer conception programs in Zimbabwe and sub-Saharan Africa.

This study has two aims: 1) to estimate the resources required (e.g., clinic visits, tests, medications, etc.) to deliver various safer conception strategies, and 2) to estimate the incremental cost per couple for “real world” scenarios for the delivery of the safer strategies in the public sector (e.g., via the Ministry of Health and Child Care [MOHCC]).

We collected data from a research clinic in Chitungwiza, Zimbabwe that specializes in delivering HIV-related care per local guidelines, with a focus on HIV prevention in at-risk populations. This is the first analysis of its kind in Zimbabwe and may inform the delivery of HIV prevention strategies in at-risk couples as a part of the broader HIV treatment and prevention national plan in African countries.

## Methods

### Overview

Within an on-going prospective research study that was providing safer conception to HIV-discordant couples in Chitungwiza, Zimbabwe (SAFER (www.clinicaltrials.gov NCT#03049176)), we measured the total cost per couple in 2017 $US ($US was the standard currency in use in Zimbabwe during data collection) for individual and combination strategies from a healthcare system perspective using micro-costing, including time & motion data. We then modeled cost for a range of implementation scenarios, representing differences from the trial in input prices, intervention intensity, and increments from current HIV prevention and treatment guidelines and practices.

### Study setting

The SAFER Study was a prospective non-randomized open-label clinical trial evaluating the uptake, adherence to, impact, and cost-effectiveness of safer reproduction strategies to prevent HIV transmission in HIV-discordant couples who have expressed a desire to conceive a child. SAFER was implemented in the University of Zimbabwe College of Health Sciences Clinical Trials Research Centre (UZCHS-CTRC) Zengeza 3 Clinical Research Site in Chitungwiza from March 2017 to July 2019. It was approved by the Medical Research Council of Zimbabwe and University of California, San Francisco ethical review boards. SAFER enrolled HIV-discordant couples (*n* = 23) who expressed a desire to conceive a child, were willing to use at least one safer strategy, and were able to provide informed consent. All counseling and services in the study were delivered per protocol, and couples received appropriate medical care and lab tests per local and global guidelines [8–12].

### Interventions

In SAFER, all couples received comprehensive HIV counselling and testing and pre-conception counseling. All couples, regardless of which partner was HIV-positive, were eligible to receive ART for the HIV-positive partner with monthly viral load testing (ART-VL) until achieving pregnancy, and quarterly viral load testing thereafter, and daily oral pre-exposure prophylaxis (PrEP, [TRUVADA®, tenofovir / emtricitabine]) for the HIV-negative partner with 6-monthly creatinine testing. Couples with an HIV-positive *male* partner were able to receive semen-washing with intrauterine insemination. Couples with an HIV-positive *female* partner were eligible to receive instruction and supplies to perform manual artificial vaginal insemination (AVI) at home. Couples selected the strategy or combination of strategies that best suited their needs and preferences. All HIV-negative partners received monthly HIV testing, and all female partners received folic acid supplementation and monthly urine pregnancy testing.

### Micro-costing of services in clinical trial

We used empirical micro-costing methods to estimate all costs associated with delivering the safer conception strategies during the trial, including personnel, consumable supplies, facilities, capital equipment (including laboratory equipment used for semen-washing and HIV and viral load testing), training, and promotion and outreach to patients [13]. We collected data on resource use and prices from SAFER Study administrative records, the UZCHS-CTRC, clinical study site, and affiliated laboratories. We tabulated total costs per strategy or combination of strategies per couple. Shared costs, such as for administration and facilities, were divided equally across couples. Because ART for HIV treatment is provided by the Ministry of Health (and not by the clinical research site), costs for ART (including personnel time, provision of ART medications, and non-safer specific tests) were estimated using published literature for Zimbabwe and the region [14, 15].

We used an estimated average time from strategy initiation to conception of 6 months based on estimates in literature [16, 17]. We did not include costs for maternity care, as these costs do not differ by strategy. We computed the cost per couple by adding the costs for inputs and services per couple – including clinic personnel time; lab tests; facility-related and infrastructure costs; and other costs, such as office and clinic supplies that are not specific to an individual strategy or related to lab tests (e.g., patient charts, cleaning solution, ovulation tracking tools) – for each individual strategy over a 6-month period, plus a 2-month “run-in” period for screening, counseling and selection of safer conception strategies. During the 2-month “run-in” period, which was prior to initiating conception attempts, all couples returned to the clinic monthly for 2 months to receive counselling on tracking menses and determining the fertile period, and counseling on safer conception options and HIV prevention; HIV negative participants received HIV antibody testing, HIV-positive participants received viral load testing; participants who opted for PrEP as safer conception were started on PrEP during the “run-in” to ensure PrEP was taken at least 3 weeks before conception attempts.

We also calculated the total cost per combination of strategies for the most commonly selected combinations. For strategy combinations, we similarly added the costs for inputs and services noted above for the 2-month run-in period and estimated 6 months of strategy use. However, because the cost of strategy combinations was less than additive due to efficiencies in the delivery of multiple strategies, services in common were counted only once to avoid double-counting. For example, costs associated with general HIV-prevention counseling (including prevention of mother-to-child transmission), counseling on ovulation tracking, pelvic exams, HIV testing for the seronegative partner, and outreach are the same regardless of the number of strategies selected, so time and resources used for these tasks and services were the same between individual strategies and strategy combinations. Additive costs, such as the incremental personnel time needed discuss more than one strategy with patients, or costs specific to a strategy (such as costs for medication, adherence counseling, and lab monitoring for PrEP when PrEP is used in conjunction with ART-VL) were accounted for in the strategy combinations.

### Personnel

We estimated personnel costs based on salary records for the types of personnel involved in delivering the safer conception strategies. SAFER Study staff are employed in the private sector; Ministry of Health salaries are used in the implementation scenarios below [18].

To quantify personnel effort to deliver each strategy, we conducted a time & motion study. Clinic staff involved in the delivery of patient services participated, including the receptionist/clerk, HIV counsellor, nurses, physicians, pharmacy staff, and onsite laboratory staff. We developed single-page forms based on previously used tools [19], with task codes specific for SAFER. We piloted the forms with staff to ensure codes were aligned with and captured all tasks. Over a typical week, clinic staff filled out one form each day as they performed SAFER-specific tasks. Using the data from the forms, we calculated the amount of staff time spent per couple for each safer conception strategy. We conducted a separate time & motion study to evaluate time spent performing offsite laboratory tests. We omitted time spent on research tasks and tasks associated with non-SAFER-Study patient care and interactions; we allocated shared time (e.g., meetings and downtime) proportionally to each strategy. Some safer-related activities occurred infrequently during the time & motion study period (eg, in-office insemination) or were not provided at the study clinic or through the SAFER Study (eg, initiation of ART due to participant demographics); time estimates for tasks related to these services were supplemented with study clinician interviews.

### Medications

Medication prices were estimated for first-line ART (TENOLAM-E, tenofovir / lamivudine / efavirenz) and oral PrEP (TRUVADA®, tenofovir / emtricitabine) from records obtained through the Zimbabwe Ministry of Health and Child Care (MOHCC). We also obtained prices from Clinton Health Access Initiative (CHAI) [20, 21] and from the UZCHS-CTRC medication price lists to gather the highest and lowest potential monthly prices for ART and PrEP for sensitivity analyses (these prices were not validated by the MOHCC). The total cost of ART and PrEP included an additional 22% overhead to drug price (per MOHCC correspondence) to capture administrative and distribution costs specific to the dispensing of ART and PrEP.

### Laboratory monitoring

We estimated laboratory test frequencies (e.g. creatinine clearance, CD4 count, HIV viral load, Hepatitis B virus) based on the SAFER protocol and guidelines [8–12]. Test costs were obtained through on- and off-site lab records for reagents and test kit prices (prorated based on the number of tests per kit or per reagent unit). Lab facility costs, lab-specific recurrent goods, and capital equipment (including overhead and operating costs based on annual costs, prorated for the number of tests run per year for each type of test) were obtained from clinic and off-site laboratory records. Lab staff costs were captured in this category and obtained through a review of lab records; interviews with lab staff, and a time & motion study.

### Other costs

Facility and infrastructure costs including rent, utilities, phone service, and water were collected from administrative records and prorated for the fraction of space within the Zengeza 3 clinical research site where the safer conception services were delivered and the number of couples enrolled in the SAFER study. Costs for non-medication recurrent goods, such as office supplies, exam gloves, cotton swabs, and cleaning supplies were estimated per couple for each type of clinic visit provided, accounting for wastage (based on clinic staff interviews).

Capital items purchased specifically for SAFER, including exam room furniture and durable clinic goods, were amortized based on estimated longevity and allocated based on estimated number of couples that could be served per year in this setting. We allocated costs associated with staff training and patient outreach based on the number of couples in the trial.

Semen-washing is not widely available in Zimbabwe; as such, we estimated startup and capital costs associated with adding semen-washing as a service within the clinical setting, amortized over a 5-year period. Data regarding capital and recurrent goods, personnel time, and other costs were collected and calculated based on delivery of semen-washing services through interviews and a review of records from a private clinic that provides these services in Harare, Zimbabwe.

### Modeling of implementation scenarios

We estimated the cost of providing safer conception services in the SAFER study as well as three real-world (non-study) implementation scenarios (Table 1). The first scenario, “High Intensity + Real-world Prices”, applies usual public sector prices to the same resource use observed in the trial. MOHCC clinics pay lower wages and have access to lower-cost reagents for viral load testing (e.g. $46.79 vs $9.40 per person, per test for reagents) [22, 23]. These lower prices are used in all three implementation scenarios. The other two scenarios also consider service intensity. The second scenario, “Target Intensity, Incremental Cost Added to Current Practice”, estimates the extra costs to deliver safer conception strategies at the target service intensity (lower than in the trial; discussed below) over and above the current standard of care in the community (e.g., ART delivery). The third scenario, “Target Intensity, Incremental Cost Added to Standard of Care”, estimates the extra costs to deliver safer conception strategies at the target service intensity that are over and above the standard of care recommended by the MOHCC, which is more intensive than current practice. Each scenario was costed by varying price and/or resource use assumptions in the micro-costing. These scenario estimation methods were based on prior cost modeling in HIV and other infectious disease programs [19, 24].

**Table 1:** Safer conception cost analysis scenarios

Scenario	Description	Components and context
**0) SAFER Study**	Total cost as **observed in the clinical trial**, including high intensity of resource use and above-market prices. **Incremental to current practice as observed in trial.**	• High private sector prices • Monthly visits and monitoring until pregnancy confirmed • Up to 12 months of conception attempts • 100% of trial participants used MOHCC delivered ART, therefore no ART cost included in this scenario • Full costs for delivery of VL testing, PrEP, AVI, and SW
**1) High Intensity + Real-world Prices**	Same resource intensity as above, but **using prices normally paid by the Zimbabwe MOHCC.**	• Public sector prices • Monthly visits and monitoring until pregnancy confirmed • Up to 12 months of conception attempts • Full costs for delivery of VL testing, PrEP, AVI, and SW
**2) Target Intensity, Incremental Cost Added to Current Practice**	Target resource intensity^a^, **incremental to current population service coverage for HIV treatment and prevention.**	• Public sector prices • After strategy initiation, visits and monitoring q3–6 months until pregnancy confirmed • Up to 12 months of conception attempts • With 72% ART coverage in community, includes costs to provide ART for 28% of HIV+, ART-naive individuals • Includes costs for needed VL tests beyond those recommended in MOHCC guidelines • Full costs for delivery of PrEP, AVI, SW, safer conception counselling
**3) Target Intensity, Incremental Cost Added to Standard of Care**	Same as above, except **incremental to current MOHCC-specified standard of care**.	• Public sector prices • After strategy initiation, visits and monitoring q3–6 months until pregnancy confirmed • Up to 12 months of conception attempts • No costs for ART and PrEP, which are in MOHCC guidelines as standard of care • Includes costs for needed VL tests beyond those recommended in MOHCC guidelines • Includes full costs for delivering AVI, SW, safer conception counselling

^a^ For a summary of target resource intensity, see Fig. 1; for a full description and definition of target resource intensity, see Supplement A

*ART* = Antiretroviral therapy. *ART-VL* = Antiretroviral therapy with frequent viral load testing. *AVI* = Artificial vaginal insemination, at home. *MOHCC* = Ministry of Health and Child Care. *PrEP* = Pre-exposure prophylaxis. *SOC* =Standard of Care.* SW* = Semen-washing

We modeled costs for an estimated average time to conception of 6 months based on literature, with a 2 month “run-in” period for screening, counseling and selection of safer conception strategies [16, 17]. Costs for 1 year of conception attempts can be made available upon request.

**Fig. 1:**
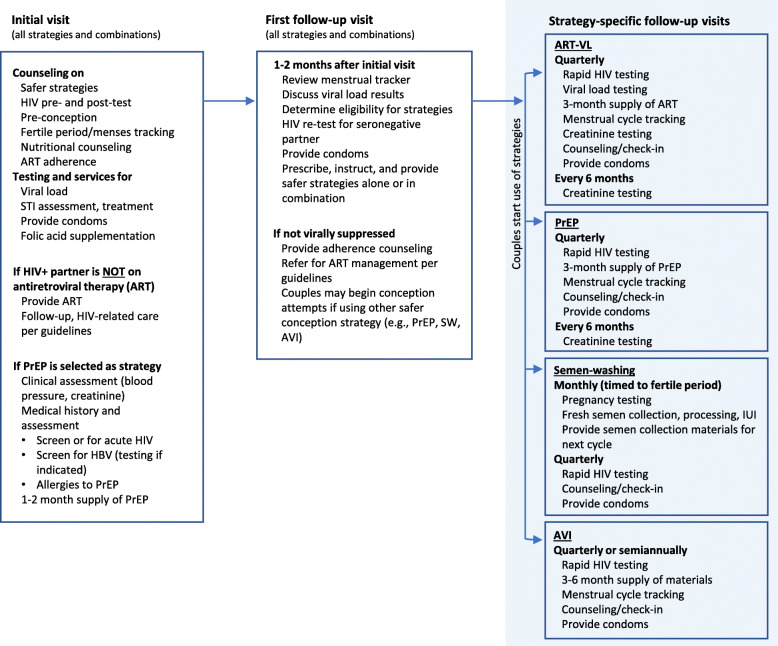
Target resource intensity and components for safer conception strategy delivery by clinic visit and safer conception strategy. ART = Antiretroviral therapy. ART-VL = Antiretroviral therapy with frequent viral load testing. AVI = Artificial vaginal insemination, at home. HBV = Hepatitis B virus. PrEP = Pre-exposure prophylaxis (with TRUVADA [emtricitabine/tenofovir disoproxil fumarate]). STI = Sexually transmitted infection

The target resource intensity for the latter two scenarios specifies the number of clinic visits, lab tests, and other resources required for the implementation of safer conception strategies in a non-research setting (Fig. 1). We interviewed local clinicians with expertise in reproductive health regarding the potential implementation of safer conception strategies in the public sector to establish a reasonable, less-intensive real-world target. The target intensity reduces costs by lowering service utilization per patient, using non-physician clinicians (such as nurses and HIV counsellors) to deliver care, and allocating fixed resources across more patients.

We based the “current practice” and “standard of care” scenarios on a review of local guidelines that govern the delivery of PrEP, ART, and other HIV prevention services and other literature regarding the delivery of HIV-related treatment and prevention in Zimbabwe [8–12]. We interviewed local experts in HIV treatment and prevention (including co-authors) to understand current standards of care and level of coverage.

### Sensitivity analyses

We also performed one-way sensitivity analyses on input variables that we observed were associated with substantial uncertainty and were not represented in the scenarios, including the price of PrEP and scale-up of the semen-washing process (i.e., increased utilization of capital equipment).

## Results

### Conception strategy selection

A total of 23 HIV-discordant couples enrolled in SAFER during the cost data collection period. Half (52%) of the couples enrolled in the SAFER Study had an HIV-positive female partner. The median age was 31 years for females (range: 21–35), 34 years for males (range: 24–54). Half (52%) of females and 70% of males completed secondary education. Half (57%) of the couples had electricity in their home. All couples were married and in monogamous relationship, and half (52%) of the couples had at least one living child together.

Twenty-two of 23 couples selected two safer conception strategies, and one couple selected three strategies. At baseline, all couples chose at least two methods: 70% chose ART-VL in combination with PrEP; 25% of couples with an HIV-positive female chose ART-VL in combination with AVI; and 27% of those with an HIV-positive male chose ART-VL in combination with semen washing. One couple chose to use a combination of ART, PrEP, and semen washing.

### Scenario input prices and Total costs

Unit prices, quantities, unit cost per couple, and data sources can be found in Supplement A; select unit prices or individual cost inputs can be found in Table 2 below. Total cost per couple (in 2017 $US) for each of the strategies and strategy combinations for the SAFER trial and for Scenarios 0, 1, 2, and 3 can be found in Table 3. In the trial (Scenario 0), individual strategies cost from $769–$1615 per couple. Implementation scenario 1 (SAFER study resource intensity but public sector prices) was one-third to two-thirds less expensive than the SAFER study costs. Under Scenario 2, which estimated the costs of delivering individual safer conception strategies using the target resource intensity (less intensive than the trial; see Fig. 1), public sector prices, and assumptions about current practice, we found the cost of delivering safer conception ranged from $73–$360 per couple. The cost of delivering the most commonly selected combination strategy, ART-VL plus PrEP, ranged from $166–$517 per couple under the three real-world implementation scenarios (Table 3).

**Table 2:** Select Unit Costs for SAFER Conception Services (2017 $US)

	SAFER Study (Observed)	Anticipated Real-world (MOHCC, Scenarios 1–3)
**Personnel (per month)**		
Physician	*	$6708.33
Nurse	*	$636.29
HIV Counsellor	*	$586.15
Pharmacist	*	$3587.21
Pharmacy tech	*	$586.15
Clerk/receptionist	*	$346.54
**Medications (per month)**		
TENOLAM-E	N/A	$9.71
PrEP (tenofovir / emtricitabine)	$49.00	$18.10
**Tests (per test)**		
HIV rapid	$16.18	$4.16
Viral load	$107.28	$25.51
CD4	$32.89	$21.43
Full Blood Count	$25.03	$7.74
Urinalysis	$3.88	$3.34
Creatinine	$4.67	$3.52
Hepatitis B	$19.37	$8.18
Urine Pregnancy	$1.80	$1.44
Syphilis RPR	$13.52	$4.19
**Administrative and overhead (per month)**		
Offsite admin & other^a^	$244.95	$3.16
Clinic supplies^b^	$14.36	$5.37
Rent, utilities & facility^c^	$404.45	$10.41
Clinic equipment (non-lab)^d^	$42.86	$3.87
Training and outreach (time and materials)	$57.08	$1.55

*SAFER Study personnel costs withheld for confidentiality. See Supplement A for total personnel costs per strategy and for additional details on all inputs and total strategy costs

^a^Includes time and costs for non-clinic, non-laboratory tasks, such as human resources, accounting, and other off-site personnel

^b^Includes consumable clinic supplies not specific to an individual strategy (paper, binders, condoms, folic acid supplements)

^c^Includes rent, maintenance, electricity, internet, phone communications, office equipment (Xerox machine and computers), security, and insurance.

^d^Includes capital goods used for clinical, non-laboratory purposes, such as an examination table, patient exam room chair, and other materials

**Table 3:** SAFER Conception total cost per couple (2017 $US)

**Individual strategies**^a^	**ART-VL**	**PrEP**	**Semen-washing**	**AVI**	
SAFER Study	$1615	$1229	$1190	$769	
High Intensity (Study-level) with Real-world Prices (Scenario 1)	$431	$403	$563	$185	
Target Intensity, Incremental Cost Added to CP (Scenario 2)	$302	$266	$360	$73	
Target Intensity, Incremental Cost Added to SOC (Scenario 3)	$132	$88	$356	$62	
**Strategy combinations**^a^	**ART-VL + PrEP**	**ART-VL + SW**	**ART-VL + AVI**	**PrEP + SW**	**PrEP + AVI**
SAFER Study	$1709	$2039	$1638	$1659	$1242
High Intensity (Study-level) with Real-world Prices (Scenario 1)	$517	$812	$444	$771	$408
Target Intensity, Incremental Cost Added to CP (Scenario 2)	$483	$695	$328	$563	$291
Target Intensity, Incremental Cost Added to SOC (Scenario 3)	$166	$450	$156	$387	$114

*ART-VL* = Antiretroviral therapy with frequent viral load testing. *AVI* = Artificial vaginal insemination, at home. *CP* = Current practice. *PrEP* = Pre-exposure prophylaxis. *SOC* = Standard of Care. *SW* =Semen-washing

^a^Total strategy costs are for 6 months of conception attempts, plus a 2-month run-in period prior to attempting conception

**Fig. 2:**
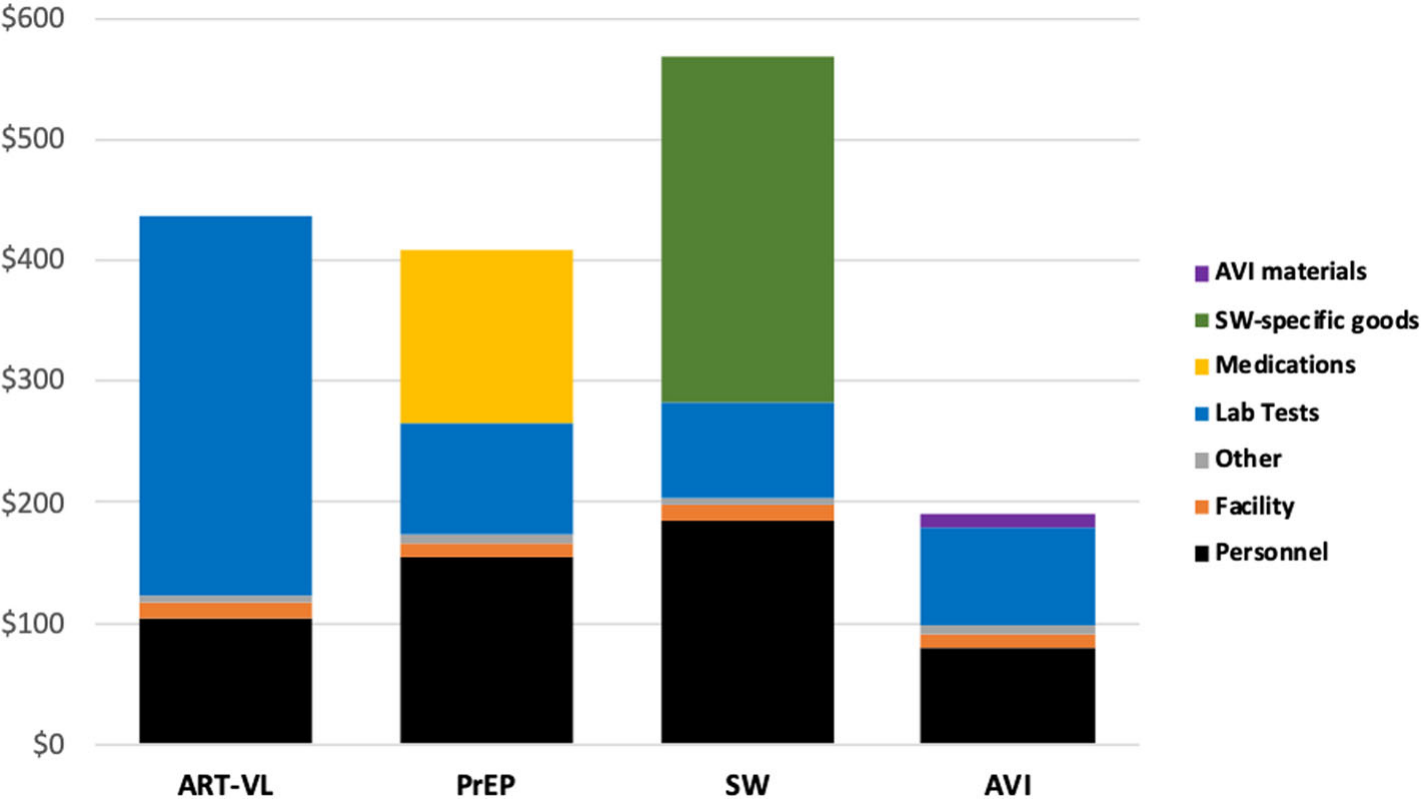
Individual strategy cost per couple (2017 $US) by input and activity type: Scenario 1 (High Intensity, Real-world Prices).ART-VL = Antiretroviral therapy with frequent viral load testing. AVI = Artificial vaginal insemination, at home. PrEP = Pre-exposure prophylaxis. SW = Semen-washing

**Fig. 3:**
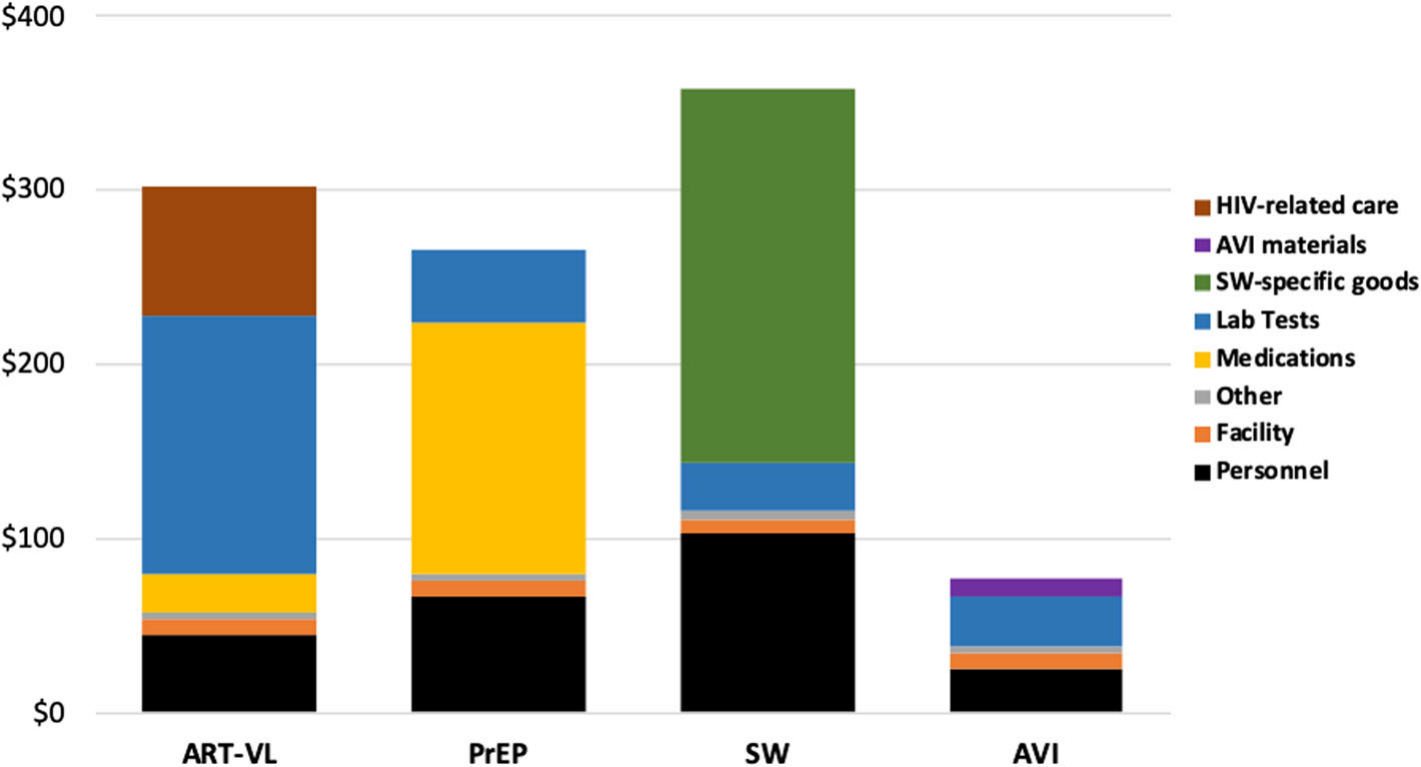
Individual strategy cost per couple (2017 $US) by input and activity type: Scenario 2 (Target Intensity, Incremental Cost Added to Current Practice). ART-VL = Antiretroviral therapy with frequent viral load testing. AVI = Artificial vaginal insemination, at home. PrEP = Pre-exposure prophylaxis. SW = Semen-washing

**Fig. 4:**
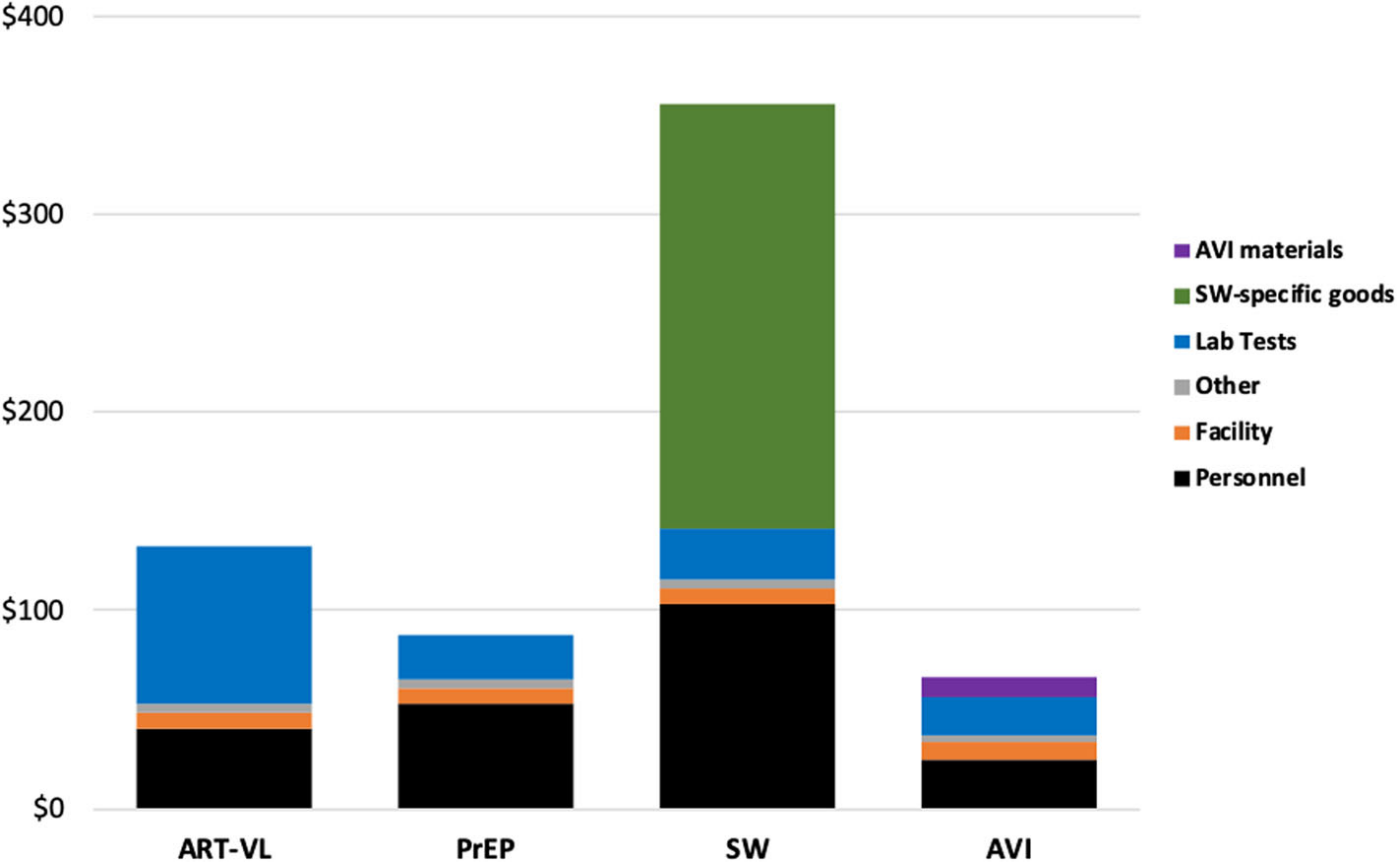
Individual strategy cost per couple (2017 $US) by input and activity type: Scenario 3 (Target Intensity, Incremental Cost Added to Standard of Care). ART-VL = Antiretroviral therapy with frequent viral load testing. AVI = Artificial vaginal insemination, at home. PrEP = Pre-exposure prophylaxis. SW = Semen-washing

Strategy cost by type of resource for each of the implementation scenarios found highest costs for personnel, lab tests, and strategy-specific goods, in highly variable proportions by strategy and scenario (Fig. 2, Fig. 3, Fig. 4).

### Variability in costs and sensitivity analyses

Results for 1-way sensitivity analyses under Scenario 2 are described in Table 4. The uncertain inputs that most affect cost are the price of PrEP, number of semen-washing attempts, and unit price per semen-washing procedure. Results of other sensitivity analyses may be made available upon request.

**Table 4:** SAFER Conception sensitivity analyses (2017 $US)

		Base Case		Adjusted range	
Strategy	Variable	Variable value	Strategy cost	Variable value	Strategy cost^a^
ART-VL	Number of VL tests	4	$302	2–8 tests	$251–$404
PrEP	Price of PrEP (per month)	$18	$266	$5.40–$49.00	$166–$513
SW	Number of attempts	3	$360	1–6 attempts	$171–$644
SW	Unit price per SW procedure	$101	$360	$84–$219	$317–$885

*ART-VL = *Antiretroviral therapy with frequent viral load testing. *AVI* = Artificial vaginal insemination, at home. *PrEP* = Pre-exposure prophylaxis. *SW* = Semen-washing

^a^Total strategy costs are for 6 months of conception attempts, plus a 2-month run-in period prior to attempting conception

## Discussion

This study estimated the costs associated with delivery of safer conception strategies in a trial and in three scenarios representing a range of real-world applications, from trial intensity but lower public prices, to reduced service intensity as expected with current practice or guidelines. Individual safer conception strategy costs are estimated to be $73–$360 per couple over and above current HIV prevention and treatment costs in this setting. The combination of safer conception strategies is more expensive, but with some efficiencies in the delivery of services that are shared by all strategies (e.g., personnel time for HIV prevention and conception counseling, HIV testing for seronegative partner, pregnancy testing). The cost of delivering the most commonly selected combination strategy, ART-VL plus PrEP, ranges from $166–$517 per couple under the three real-world implementation scenarios. Thus, programmatic context is extremely important. Major drivers of differences in cost across strategies and scenarios include the frequency and intensity of clinic visits, lab tests, and procedures; population coverage of anti-retroviral medications and HIV-related care; price and availability of PrEP; scale-up of semen-washing and number of semen-washing attempts needed per couple; the use of a combination of safer conception strategies versus a single strategy; private- versus public-sector pricing for goods and personnel; and type of personnel delivering services (e.g. physician or medical officer versus nurse or HIV counsellor.

We employed an intensive approach to cost data collection, including a time and motion study and microcosting to portray differences in resource utilization (total and components) among strategies. We also modeled different implementation scenarios to extrapolate our trial findings to the real world.

Though allowing couples to select a combination of strategies led to higher per-couple costs in this study, offering couples a range of strategies and allowing them to choose the options that best meet their needs may help improve adherence [25, 26]. In turn, improved adherence to HIV prevention methods has been demonstrated to significantly increase the efficacy of interventions in several major studies [27–29]. Furthermore, the most commonly selected strategy was ART with frequent viral load testing; promoting the use of ART among HIV-positive individuals not only reduces the risk of transmission to others, it also helps improve the health and wellbeing of the index partner [30].

This study has limitations. First, the SAFER trial had just one research site and a relatively small sample size. While this may limit generalizability, the unit costs we found were consistent with those reported in literature in similar settings in the region [31–34]. Second, our estimation of costs associated with scenarios of safer strategy delivery in the public sector are speculative, even if based on data from published literature and local experts. Some costs collected in this study reflect costs associated with private sector-delivered care, which may result in higher cost estimates than in the public sector. We worked to mitigate these issues by combing literature for additional data and working with local experts to compare the resources and costs in this analysis with those in the public sector.

Third, our scenarios that include ART-VL rely on published estimates of the resources and costs to deliver HIV-related care that are not specific to safer conception (e.g. cost of ART, ART-specific clinic visits, etc.) and were not delivered in the trial. However, the costs for HIV-related care that we used are from the region [14, 15, 31]. Finally, we calculated costs for safer conception strategies using a mix of input-based and activity based-costing. While we took care to not double-count, this method may obscure the total overall costs associated with personnel and facilities, for example, as lab-specific costs include personnel and facility costs for all lab activities.

Lastly, since this study is from a healthcare system perspective, it does not include patient/client-level costs, such as those associated with travel to and from clinics or for productivity losses associated with receiving and using the safer strategies. The “real-world” scenarios in our study assume that – because of the MOHCC’s commitment to covering medical costs associated with the treatment and prevention of HIV, especially in high-risk groups – the MOHCC would incur all clinical costs associated with the delivery of these safer strategies. If a significant cost burden were placed on couples, it may impact selection of strategies, uptake, and adherence to these strategies. Since adherence to HIV treatment and prevention measures is critical to achieving national and global goals, costs to couples and the healthcare system should be carefully considered [27–29].

## Conclusions

To our knowledge, this is the first study to analyze the costs associated with the delivery of individual and combined safer conception strategies for HIV prevention in discordant couples who wish to conceive a child. While these strategies have been demonstrated to help serodiscordant couples conceive a child, they are not yet widely available in Zimbabwe and many other low-resource settings. Cost data may help implementers and policymakers add safer services to the resources they offer to couples impacted by HIV.

To better understand both the cost and health impact of delivering the safer strategies in Zimbabwe, we are undertaking further analyses to explore the clinical outcomes, acceptability, and modelling of the cost-effectiveness of individual and combination strategies compared to no safer conception strategies. Integrating the safer strategies into current policy and practice in Zimbabwe would require financial investment and commitment among health authorities and HIV care providers. However, in our cost modeling, we found that the incremental resources and costs associated safer conception services – especially for ART-VL and PrEP – are complementary to the treatment and prevention services already included in Zimbabwe’s HIV guidelines. Semen-washing and artificial vaginal insemination at home offer additional options for couples who would prefer these methods either individually or in combination with other strategies.

Successful HIV prevention among discordant couples who would like to conceive a child has the potential to avert new infections in two generations and can help achieve the UNAIDS goal of getting to zero new infections by 2030.

## Supplementary information

**Additional file 1.**

## Data Availability

Data generated or analyzed in this study are included in this published article or in the supplementary file (Supplement A: SAFER Cost Analysis). This supplement includes unit prices, quantities, unit cost per couple, and data sources for the safer conception strategies and strategy combinations included in this analysis. For the purposes of confidentiality, some data regarding costs from the SAFER Study have been withheld and some workbook sheets are protected, however, data are available from the corresponding author on reasonable request.

## Abbreviations

ART: Antiretroviral therapy
ART-VL: Antiretroviral therapy with frequent viral load testing
AVI: Artificial vaginal insemination
CP: Current practice
PrEP: Pre-exposure prophylaxis
HIV: Human immunodeficiency virus
IUI: Intrauterine insemination
MOH: Ministry of health
MOHCC: Ministry of health and child care
PrEP: Pre-exposure prophylaxis
SOC: Standard of care
SW: Semen-washing
TENOLAM-E: Tenofovir / lamivudine / efavirenz
UCSF: University of California, San Francisco
UZCHS-CTU: University of Zimbabwe college of health sciences clinical trials unit
